# Dexpanthenol in Wound Healing after Medical and Cosmetic Interventions (Postprocedure Wound Healing)

**DOI:** 10.3390/ph13070138

**Published:** 2020-06-29

**Authors:** Julian Gorski, Ehrhardt Proksch, Jens Malte Baron, Daphne Schmid, Lei Zhang

**Affiliations:** 1 Bayer Vital GmbH, Building K 56, D-51368 Leverkusen, Germany; julian.gorski@bayer.com; 2 Department of Dermatology, University of Kiel, Schittenhelmstrasse 7, D-24105 Kiel, Germany; eproksch@dermatology.uni-kiel.de; 3 Department of Dermatology and Allergology, RWTH Aachen University, Pauwelsstrasse 30, D-52074 Aachen, Germany; jbaron@ukaachen.de; 4 Bayer Consumer Care AG, Peter Merian-Strasse 84, CH-4002 Basel, Switzerland; daphne.schmid@bayer.com

**Keywords:** wound healing, dexpanthenol, postprocedure, minor wounds

## Abstract

With the availability of new technologies, the number of subjects undergoing medical and cosmetic interventions is increasing. Many procedures (e.g., ablative fractional laser treatment) resulting in superficial/minor wounds require appropriate aftercare to prevent complications in wound healing and poor cosmetic outcome. We review the published evidence of the usefulness of topical dexpanthenol in postprocedure wound healing and the associated mechanisms of action at the molecular level. A search in the PubMed and Embase databases was performed to query the terms dexpanthenol, panthenol, superficial wound, minor wound, wound healing, skin repair, and postprocedure. Search results were categorized as clinical trials and in vitro studies. In vitro and clinical studies provided evidence that topically applied dexpanthenol promotes superficial and postprocedure wound healing. Latest findings confirmed that dexpanthenol upregulates genes that are critical for the healing process. The gene expression data are of clinical relevance as evidenced by prospective clinical studies indicating that topical dexpanthenol accelerates wound healing with rapid re-epithelialization and restoration of skin barrier function following skin injury. It can therefore be inferred that topical dexpanthenol represents an appropriate and state-of-the-art treatment option for superficial postprocedure wounds, especially when applied early after the superficial skin damage.

## 1. Introduction

Many medical and cosmetic interventions, such as ablative laser treatment, dermabrasion, microneedling, or tattooing, result in superficial/minor wounds; this affects the integrity of the epidermis and requires postprocedural wound care to assure a proper healing process of the damaged skin [1,2,3]. Topical compounds that support all three phases of wound healing (inflammation, proliferation, and remodeling) are considered particularly useful for the treatment of minor and superficial wounds [4]. Recently, it has been suggested that dexpanthenol exhibits activity across all three wound healing phases [4].

Dexpanthenol is well absorbed when applied topically to the skin and rapidly converted to pantothenic acid [5,6,7]. The latter is a coenzyme A constituent and essential for the physiological function of epithelia [8,9]. Dexpanthenol supports skin regeneration by enhancing epidermal differentiation and facilitates wound healing [9,10,11]; it also showed activity in the prevention of biofilm formation and has anti-inflammatory effects [12,13]. Furthermore, dexpanthenol acts as a moisturizer and skin barrier enhancer [14,15,16,17]. In dry skin conditions, it compensates for reduced hydration by increasing water content and by beneficially influencing the molecular mobility of the stratum corneum lipid lamellae and proteins [9,11,18]. These features triggered the development of various topical dexpanthenol-containing galenical formulations which are widely used in the dermatological field. Topical dexpanthenol has also been recommended for the treatment of minor and superficial wounds [9].

In this paper, we review the usefulness of topical dexpanthenol in postprocedure wound healing and the associated mechanisms of action at the molecular level.

## 2. Search Strategy

A systematic literature search was performed in the PubMed and Embase databases. Various combinations of the following terms were queried: dexpanthenol, panthenol, superficial wound, minor wound, wound healing, skin repair, postprocedure. In addition, references cited in related publications were followed up. Articles for this review were restricted to clinical trials (in vivo data) and in vitro studies. There were no date or language criteria for inclusion in this paper. In total, the search identified 33 publications. Of these, 8 articles were of relevance for assessing the usefulness of topical dexpanthenol in wound healing after medical and cosmetic interventions. The most frequent reasons for article rejection were studies involving animals and trials investigating dexpanthenol combination products.

## 3. Types of Wounds and Process of Repair

### 3.1. Wounds

Wounds can be classified as acute or chronic. Chronic wounds frequently occur in patients with underlying diseases (e.g., diabetes or malignancies), are difficult to heal, and need special wound management [19]. Acute wounds are most frequently caused by mechanical injuries but can also be caused by burns or chemical harms [19]. The management of this type of wound is performed on an individual basis and varies depending on the wound location and characteristics [20,21]. Acute wounds may involve deeper structures of the dermis (including blood vessels, sweat glands, and/or hair follicles) which are referred to as partial-thickness wounds. In the case of full-thickness wounds, the subcutaneous fat or deeper tissues are additionally damaged [19,22]. In superficial wounds of the skin (e.g., abrasions or superficial thermal wounds), the epidermis is affected [19]; superficial parts of the dermis may be involved as well [22]. The term “minor wounds” usually describes small acute cutaneous wounds, such as small cuts, scraps, fissures, or superficial incisions [22,23]. If bleeding from minor wounds occurs, it can be stopped by putting pressure on the affected skin area or by using dressings or skin tapes. Frequently, subjects treat superficial/minor everyday wounds themselves [24].

Superficial and minor wounds also occur following scheduled medical or cosmetic interventions, such as ablative laser treatment of the skin (ablative laser skin resurfacing), dermabrasion, or the tattooing procedure. During ablative laser therapy, the epidermis and parts of the dermis are removed while non-ablative laser therapy leaves the skin surface undamaged [25,26]. A more recently applied laser technique for skin treatments is fractional laser. Fractional lasers deliver the laser light in fractions onto the skin surface and work either ablative or non-ablative [25,27]. Following ablative fractional laser treatment, ablation zones (holes) are present in the epidermis and superficial dermis that are surrounded by areas with intact skin surface [25,28]. In response to all these laser therapies, overheating of epidermal and dermal compartments may occur which can cause thermal injury, especially after CO_2_ laser irradiation [25,26,29].

The use of ablative lasers has been established as an effective technique to treat aged or sun-damaged skin (e.g., actinic keratosis [30]). Frequently used lasers are CO_2_ and erbium-doped yttrium aluminum garnet (Er:YAG) lasers [26]. For ablative fractional laser treatments, CO_2_ and Er:YAG lasers are both applied, but the CO_2_ laser is generally preferred by dermatologists and has become the method of choice in the therapy of photo-damaged skin [2,25,31].

During a tattooing session, the tattoo needle punches repeatedly through the epidermis and introduces pigments and dyes into the dermis; generally, the skin injuries are confined to the epidermis and upper parts of the dermis depending on the experience of the tattooing person [32,33,34]. In parallel to the increasing popularity of tattoos, the number of subjects who would like to get them removed has also increased. For tattoo removal, laser treatment has become the gold standard [35,36]. The laser selection depends on the tattoo colors and skin type, but the Q-switched YAG laser is considered the standard laser therapy for tattoo removal [36,37]. Particularly for multicolored tattoos, ablative fractional laser treatment (alone or in combination with other laser techniques) might be an alternative option for effective tattoo removal [25,35].

Selective absorption of the laser light by the pigments results in fragmented tattoo particles which allows transportation of the tinier fragments away from the skin via phagocytosis [36,38]. Tattoo removal by lasers typically causes postprocedure open wounds on the treated skin due to the high energy of the intense light pulses [38].

### 3.2. Wound Healing

All the aforementioned superficial or minor wounds require proper care to achieve a scar-free wound healing or healing with a subtle scar only [22,39]. Although the skin injuries are minor in nature, a proper and early wound care is advised to accelerate re-epithelization and thus minimize infection risk and the formation of a hypertrophic scar [24]. Even if the injury occurred in the context of a scheduled and controlled intervention with adequate hygiene measures, there is the necessity of topical postprocedure wound care; it has been shown that effective postprocedure wound healing reduces the incidence of postprocedure complications which ensures a satisfying cosmetic outcome as much as possible [40,41]. An undisturbed wound healing results in a better cosmetic result. Consequently, an appropriate postprocedure topical wound treatment has been recommended after ablative laser treatments of the skin [2]. Likewise, following the tattooing procedure, it has been emphasized that aftercare is actually wound care [42]. If performed inadequately, there is an increased risk of infection and wound healing may be impaired. In addition, it could have a negative impact on the aesthetic outcome of the tattoo procedure [1]. Similarly, in case the tattoo will be removed by laser techniques, the application of ointments and protective dressings are recommended [35].

Wound healing is a dynamic process and represents the body’s response to injury [43,44,45]. The aim of wound healing after minor injury is to restore skin integrity in a timely fashion with an appearance and functionality which ideally is indistinguishable from the preinjury skin [4]. Acute superficial/minor cutaneous wounds usually heal within three weeks after which barrier function and normal skin structure are fully restored, although formation of scars may occur [22,46]. Traditionally, the wound healing process has been divided in three sequential phases: inflammation, proliferation, and matrix deposition (remodeling) [47,48]. Recent research on the different steps involved in wound healing suggests that these three phases of wound repair overlap with multiple actions running in parallel [4].

A three-dimensional (3D) human full-thickness skin model was developed to study the morphological and molecular alterations during the healing process of epidermal wounds [3,49,50]. The 3D skin model consists of epidermal and dermal layers comprising a functional stratum corneum, basal layer, and basal membrane [3,51]. Although this in vitro model cannot fully mimic the complex in vivo conditions, it is able to provide insight into the histological and molecular effects induced by superficial injuries [3]. By means of the 3D skin model, the histological changes and shifts in gene expression related to epidermal differentiation, inflammation, and remodeling were defined that occur after epidermal wounding caused by ablative fractional Er:YAG laser, ablative fractional CO_2_ laser, or microneedling [3,50,51]. After microneedling, genes like COL3A1, COL8A1, and TIMP3 were upregulated whereas pro-inflammatory cytokines were downregulated [3]. Following ablative fractional Er:YAG laser treatment, there was an increased mRNA expression of matrix metalloproteinases and their inhibitors (e.g., MMPs), chemokines (e.g., CXCL1 and CXCL2), and cytokines (e.g., IL6, IL8, and IL24), whereas mRNA expression of epidermal differentiation markers (e.g., keratin-associated protein 4, filaggrin 1, and filaggrin 2) was reduced [51]. The findings allow studying the pharmacological effects of topical treatments in these settings as a substitute for in vivo studies [51]. Data gathered with the 3D skin model showed good correlations with data retrieved from human skin in vivo studies [49,52,53]. Both laser-irradiated human 3D skin cultured in calcium pantothenate-containing medium and samples of dexpanthenol-treated injured human skin showed a decreased expression of S100A7 (psoriasin), a protein detected in epidermal layers of acute and chronic wounds [49,53]. Recently, also a human full-thickness 3D non-keratinized mucous membrane model has been established [54]. It mimics two layers of the mucous membrane (stratified squamous epithelium and lamina propria) and allows studying effects of topical agents on mucosa histology and gene expression.

## 4. Dexpanthenol in Wound Healing

Wound management has to simultaneously address all three phases of wound healing and should comprise protection from infection and free radicals, modulation of inflammation, support of cell proliferation, and acceleration of migration [4]. Baron et al. recently suggested that dexpanthenol fulfills these criteria (with the caveat that for infection protection a mild antiseptic has to be added), thereby exhibiting activity across all three wound healing phases [4]. In fact, dexpanthenol was shown to assist the different steps of wound-healing in various in vitro and in vivo studies [4,9]. Specifically, topically applied dexpanthenol-containing formulations facilitated wound healing in in vivo models of superficial skin [55,56,57] as well as in women with nipple cracks and fissures [58,59,60], and were used as reference preparation in trials investigating new developments for the care of minor everyday wounds [61].

### 4.1. Role of Dexpanthenol on Postprocedure Wound Healing—In Vitro Data

Marquardt and colleagues studied the effects of dexpanthenol on wound healing and gene regulation by employing the 3D skin model [49]. Skin equivalents were irradiated with a non-sequential fractional ultrapulsed CO_2_ laser. Subsequently, the injured skin equivalents were treated for three days with various 5% dexpanthenol-containing ointments or petroleum jelly (vaseline). Laser irradiated untreated 3D skin served as control. Histological, microarray, and quantitative reverse transcription polymerase chain reaction (qRT-PCR) analyses were performed at different time points. Topical treatment of skin wounds with 5% dexpanthenol formulations enhanced wound closure compared with the CO_2_ laser-irradiated untreated control or wounds treated with petroleum jelly. Culture analyses confirmed that the observed favorable effect was caused by dexpanthenol and not by the ointment base. Culturing laser-irradiated skin equivalents in calcium pantothenate-containing medium resulted in different molecular effects. Among them, gene expressions of interleukin (IL)-1α, a proinflammatory cytokine that acts across the three wound healing phases [4], and matrix metalloproteinase 3 (MMP3), a regulator of wound healing [62], were upregulated. Moreover, three days after skin injury, immunofluorescence studies showed an upregulated Ki67 protein expression in laser-irradiated 3D skin cultured with calcium pantothenate compared to control. The results are noteworthy because, without treatment, the expression of IL-1α and MMP3 is actually downregulated following fractional ultrapulsed CO_2_ laser irradiation [50]. The upregulation of the Ki67 protein, a proliferation marker, is in accordance with an earlier study which used an in vitro model of artificially wounded monolayers (scrape wounds) [52]. There, dermal fibroblasts, incubated with calcium pantothenate (20 µg/mL), revealed an enhanced proliferation compared with untreated cells, suggesting a stimulatory effect of pantothenate on the proliferation of dermal fibroblasts. Among others, genes coding for IL-6 and IL-8 were upregulated by calcium pantothenate. Both cytokines are important for the healing of human dermal wounds [9,63].

**Figure 1:**
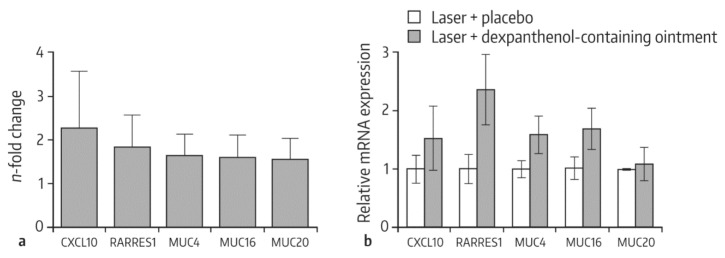
Gene expression profiling in 3D mucous membrane models after laser injury and treatment with a dexpanthenol-containing ointment in comparison with the placebo-treated control. (**a**) 3D mucous membrane models were harvested 3 days after laser treatment (100 mJ/cm^2^), and gene expression was measured using the Affymetrix^®^ Gene Chip Human Exon 2.0 ST microarray. Results of 3 experiments were pooled. (**b**) TaqMan real-time polymerase chain reaction (qRT-PCR) analysis of selected genes on day 3 after treatment. Mean values with standard error of the mean (SEM) of 3 independent experiments performed in duplicate are shown. From Ref. [54] with kind permission from S. Karger AG (www.karger.com/SPP).

In another study, Schmitt and coworkers investigated the molecular effects of dexpanthenol on mucosal wound healing after introducing standardized lesions with a non-sequential fractional ultrapulsed CO_2_ laser [54]. For that purpose, the novel human full-thickness 3D non-keratinized mucous membrane in vitro model was employed. Immediately after laser injury, models were topically treated with an ointment containing 5% dexpanthenol or placebo for 5 days. The culture medium was free of dexpanthenol or other ingredients known to increase proliferation. Histological examinations led to the conclusion that treatment with the dexpanthenol-ointment enhanced wound closure compared with placebo. Gene expression analyses revealed that topical application of dexpanthenol was associated with a >1.5-fold upregulation of various genes involved in wound healing, such as the CXCL10 gene, mucin protein family genes, and the retinoic acid receptor responder protein 1 gene (RARRES1) (Figure 1).

### 4.2. Role of Dexpanthenol on Postprocedure Wound Healing—In Vivo Data

Girard et al. conducted a double-blind, intra-individual comparative trial that studied the postprocedure wound healing effects of an ointment containing 5% dexpanthenol [64]. In total, 35 patients received an autologous skin graft because of burns. For 14 days, the dexpanthenol-containing formulation or vehicle was topically applied to the mesh graft donor sites (i.e., to sites from which skin grafts were removed). These superficial wounds tended to heal more rapidly at the sites treated with dexpanthenol than at the skin areas treated with vehicle as assessed clinically and by measurements of microcirculation, temperature, and biomechanical properties. During the second week of the study, the skin areas topically treated with dexpanthenol were more hydrated than those treated with vehicle (*p* = 0.05) based on clinical scores. Moreover, pruritus related to wound healing resolved sooner (*p* = 0.06).

A randomized, double-blind study in healthy subjects investigated the modulation of gene expression by dexpanthenol in previously injured punch skin biopsies [53]. In each subject, two minor wounds were introduced by injuring the skin with 4 mm punch biopsies. One wound was topically treated with a 5% dexpanthenol-containing ointment and one wound with placebo. Eight-millimeter punch biopsies of dexpanthenol- and placebo-treated skin areas were taken up to 144 h following treatment initiation. Subsequently, the biopsy material was analyzed to compare the postprocedure gene expression profile of dexpanthenol-treated wounds in comparison with placebo-treated wounds. In specimens treated with dexpanthenol, upregulation of genes involved in wound healing was observed (i.e., IL-6, IL-1β, CYP1B1, CXCL1, CCL18, and KAP 4-2).

**Figure 2:**
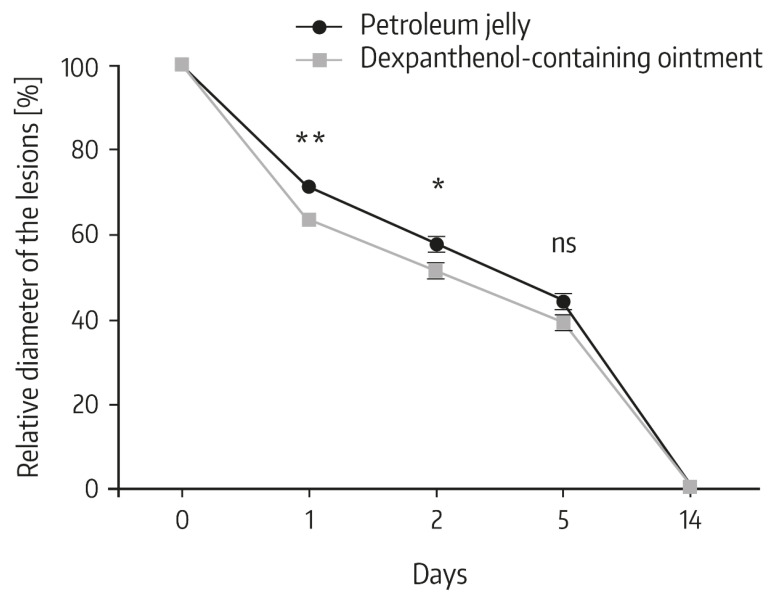
Change of the diameter of the individual lesions between study visits. The diameter of 10 point lesions was determined immediately after laser therapy and mean diameter was set to 100% and used for standardization. Data show mean ± standard deviation with *n* = 38, * *p* < 0.05, ** *p* < 0.01 (Mann–Whitney *U* test); ns: not significant. From Ref. [2] reprinted with permission of Informa UK Limited, trading as Taylor & Francis Group (www.tandfonline.com).

**Figure 3:**
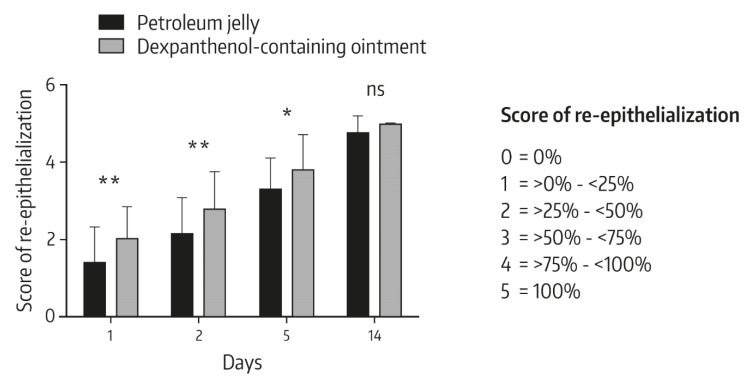
Visual changes of the wound healing between study visits. The wound healing rates were visually assessed, based on the measure of re-epithelialization. Data show mean ± standard deviation with *n* = 38, * *p* < 0.05, ** *p* < 0.01 (Mann–Whitney *U* test); ns: not significant. From Ref. [2] reprinted with permission of Informa UK Limited, trading as Taylor & Francis Group (www.tandfonline.com).

Heise et al. performed a randomized, controlled 14 day in vivo study to compare the effects of a 5% dexpanthenol-containing ointment with petroleum jelly on wound healing after fractional ablative CO_2_ laser treatment of photo-damaged skin [2]. In total, 38 patients participated in the study. In each patient, the wound surface was divided in two parts following laser treatment. One part was treated with the dexpanthenol-containing ointment and one area was treated with petroleum jelly for a duration of 7 days. In the first days of treatment, the topical use of dexpanthenol was associated with faster wound healing than with petroleum jelly. Specifically, on days 1 and 2, lesions treated with the dexpanthenol-containing ointment showed a significantly smaller diameter compared to the original diameter than lesions treated with petroleum jelly (Figure 2). Similarly, the degree of re-epithelialization was significantly greater in wounds topically treated with dexpanthenol on days 1, 2, and 5 (Figure 3), as were visual analog scale (VAS) scores for cosmetic appearance.

**Figure 4:**
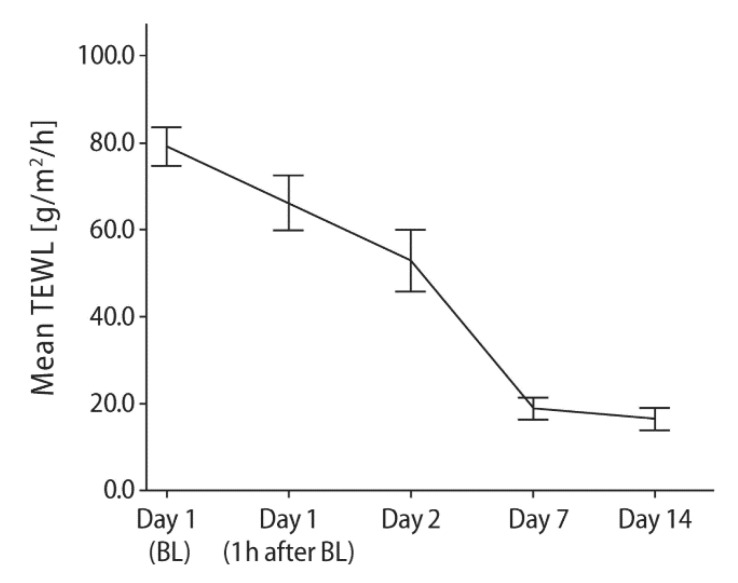
Mean (±95% confidence interval) TEWL following topical application of an ointment containing 5% dexpanthenol on freshly tattooed skin 4–8 times daily for 14 days. BL = Baseline assessment at approximately 4 h after the tattooing session and immediately before treatment initiation. TEWL = transepidermal water loss. From Ref. [34] with kind permission from Wounds International (www.woundsinternational.com).

Another prospective, investigator-blinded, intra-individual comparison study investigated the effects of two 5% dexpanthenol water-in-oil formulations (ointment and emulsion) on freshly tattooed skin in healthy subjects having received two new tattoos of comparable size [34]. The dexpanthenol formulations were applied 4–8 times daily for 14 days, starting at 4 h after the tattooing session. Primary outcome variable was change in transepidermal water loss (TEWL) over time. A reduction of TEWL reflects improvement in skin barrier function, which is primarily located in the stratum corneum [65,66]. In total, 54 subjects underwent repeated TEWL measurements. The tattooing procedure induced a 7-fold increase in mean TEWL (i.e., a significant barrier dysfunction). The topical use of both 5% dexpanthenol water-in-oil formulations was associated with a pronounced reduction in TEWL over the study period without apparent differences between formulations. Table 1 and Figure 4 show the results for the ointment. With both formulations, there was a virtually complete restoration of skin barrier function after 14 days of treatment which was 1 week earlier than previously reported for untreated sodium dodecylsulfate solution (SDS) challenged skin [16]. This is noteworthy because tattooing is more traumatic to the skin than topical application of a skin irritant (SDS). In all subjects, an uncomplicated healing process was observed and postprocedure cosmetic performances were highly rated by study participants.

### 4.3. Role of Galenic Composition on Postprocedure Wound Healing

Procedures resulting in superficial/minor wounds require an appropriate aftercare to assure expedited re-epithelialization and low infection risk [1,2]. In this setting, ointments are frequently utilized. They form a semi-occlusive breathable film that protects the wound from external influences (e.g., pathogens or contaminants), keeps the injured area hydrated but avoids moisture congestion, and supports a successful skin barrier restoration [1,24]. For the care of epidermal wounds, an air interface and appropriate partial pressure of oxygen are considered important for achieving rapid re-epithelialization [22,67]. It has been suggested that semi-occlusion of epidermal injuries results in a superior epidermal response and an earlier achievement of skin barrier function compared to an occlusive wound management [67]. It can therefore be inferred that an ointment is a suitable and still modern galenical formulation for postprocedure wound healing.

Historically, a 5% dexpanthenol-containing ointment has been used by tattooists in the postprocedure wound care despite the fact that supporting studies in this setting were lacking for many years [68]. Recently, clinical data were generated which provide scientific evidence for the use of a 5% dexpanthenol ointment in the wound aftercare of freshly tattooed skin [34]. For the wound care following ablative laser treatments of the skin, petroleum jelly is currently recommended by the manufacturers of ablative laser systems until encrustation of the affected skin area decreases [2]. However, petroleum jelly is a wax-like, difficult-to-handle material, particularly when larger skin areas have to be covered [69]. In addition, petroleum jelly has rather strong occlusive effects following topical application. In fact, it is used as positive reference product when the occlusion properties of new topical galenical developments are studied [69]. Results from a recent head-to-head comparative trial provided evidence that a 5% dexpanthenol-containing ointment is superior to petroleum jelly in the wound care after fractional ablative laser treatment as reflected by a faster wound closure and higher re-epithelialization rate as well as better cosmetic outcomes [2].

Today, different topical dexpanthenol formulations exist (cream, emollient, drops, gel, lotion, oil, ointment, solution, spray), developed to meet individual requirements [9]. There is cumulative evidence, that out of this product range, the ointment is a suitable option for postprocedure wound care.

### 4.4. Future Perspectives

With the availability of new treatment techniques, it can be expected that in the near future, there will be an increased need for postprocedural care of superficial/minor wounds and thus topical semi-occlusive formulations accelerating re-epithelialization and healing of the injured skin. Particularly the recent insights in the potential of skin treatments with innovative lasers opened the door for a variety of avenues to be pursued [25]. Apart from the use of ablative lasers in the treatment of UV-damaged, pre-cancerous skin areas (e.g., actinic keratosis), promising results have been obtained in the treatment of subjects with severely wrinkled skin [25], advanced photoaging of the face [70], and atrophic scars [71,72]. Ablative lasers might also be beneficial for the therapy of severe rhinophyma [73], burn scars [74], and other conditions. This type of treatment concept could also be extended to other epithelia, such as affected mucosal membranes (e.g., leukoplakia) [25]. It has been suggested that the temporary opening of the epidermal barrier reflects one of the greatest potentials of ablative fractional lasers [25]. With this concept, there is the opportunity for a simplified and intensified delivery of compounds into the dermis and epidermis. Further research is ongoing in this field.

## 5. Conclusions

Medical and cosmetic interventions resulting in superficial or minor wounds require appropriate aftercare to prevent complications in wound healing (e.g., infection or scar formation) and to restore skin integrity with an appearance and functionality which ideally is indistinguishable from the preinjury skin. Particularly, an expedited re-epithelialization and restoration of skin barrier function are considered key for an uneventful healing process. In this setting, ointments forming a semi-occlusive breathable film and providing a moist wound environment facilitate healing. Cumulative evidence from various in vitro and in vivo studies provided scientific evidence that a dexpanthenol-containing ointment is—beyond the care of everyday wounds—a suitable option for postprocedure wound care.

With the availability of novel skin models, new insights could be gained on the way dexpanthenol influences the healing process of epidermal wounds at the molecular level. Gene expression data from in vitro studies using the 3D skin model suggest that dexpanthenol upregulates genes that are critical for wound healing. Gene expression data gathered with the 3D skin model showed good correlations with gene expression profiles observed in in vivo studies with dexpanthenol. Moreover, the gene expression data from both in vitro and in vivo studies are of clinical relevance as evidenced by prospective clinical studies indicating that dexpanthenol promotes wound healing with rapid re-epithelialization and restoration of skin barrier function following skin injury. In a comparative study in the setting of fractional ablative laser treatment, the dexpanthenol-containing ointment revealed superior re-epithelialization rates and better cosmetic outcomes in comparison with standard treatment (petroleum jelly). It can therefore be concluded that topical dexpanthenol represents an appropriate and modern treatment option for postprocedure wounds, especially when applied early after the superficial skin damage.

## Figures and Tables

**Table 1:** Mean change from baseline in transepidermal water loss (TEWL) following application of 5% dexpanthenol-containing ointment on freshly tattooed skin 4–8 times daily over 14 days *.

Time	Change of TEWL	*p*-Value ^#^
Day 1 (BL)	79.14 ± 15.98	-
Day 1 (1 h after BL)	−12.99 ± 19.25	0.001
Day 2	−26.16 ± 25.30	<0.001
Day 7	−60.23 ± 17.35	<0.001
Day 14	−62.62 ± 18.39	<0.001

*n* = 54. Data are given in g/m^2^/h. All values are presented as mean ± standard deviation. BL = Baseline assessment = Mean TEWL value at approximately 4 h after the tattooing session and immediately before first application of the ointment. TEWL = transepidermal water loss. * modified from Ref. [34]. ^#^ Dunnett’s two-tailed *t*-test for change from baseline.
